# The Challenges and Pitfalls of Detecting Sleep Hypopnea Using a Wearable Optical Sensor: Comparative Study

**DOI:** 10.2196/24171

**Published:** 2021-07-29

**Authors:** Zhongxing Zhang, Ming Qi, Gordana Hügli, Ramin Khatami

**Affiliations:** 1 Center for Sleep Medicine Sleep Research and Epileptology Barmelweid Switzerland; 2 Barmelweid Academy Clinic Barmelweid AG Barmelweid Switzerland; 3 Department of Neurology Bern University Hospital and University of Bern Bern Switzerland

**Keywords:** obstructive sleep apnea, wearable devices, smartwatch, oxygen saturation, near-infrared spectroscopy, continuous positive airway pressure therapy, photoplethysmography

## Abstract

**Background:**

Obstructive sleep apnea (OSA) is the most prevalent respiratory sleep disorder occurring in 9% to 38% of the general population. About 90% of patients with suspected OSA remain undiagnosed due to the lack of sleep laboratories or specialists and the high cost of gold-standard in-lab polysomnography diagnosis, leading to a decreased quality of life and increased health care burden in cardio- and cerebrovascular diseases. Wearable sleep trackers like smartwatches and armbands are booming, creating a hope for cost-efficient at-home OSA diagnosis and assessment of treatment (eg, continuous positive airway pressure [CPAP] therapy) effectiveness. However, such wearables are currently still not available and cannot be used to detect sleep hypopnea. Sleep hypopnea is defined by ≥30% drop in breathing and an at least 3% drop in peripheral capillary oxygen saturation (Spo_2_) measured at the fingertip. Whether the conventional measures of oxygen desaturation (OD) at the fingertip and at the arm or wrist are identical is essentially unknown.

**Objective:**

We aimed to compare event-by-event arm OD (arm_OD) with fingertip OD (finger_OD) in sleep hypopneas during both naïve sleep and CPAP therapy.

**Methods:**

Thirty patients with OSA underwent an incremental, stepwise CPAP titration protocol during all-night in-lab video-polysomnography monitoring (ie, 1-h baseline sleep without CPAP followed by stepwise increments of 1 cmH_2_O pressure per hour starting from 5 to 8 cmH_2_O depending on the individual). Arm_OD of the left biceps muscle and finger_OD of the left index fingertip in sleep hypopneas were simultaneously measured by frequency-domain near-infrared spectroscopy and video-polysomnography photoplethysmography, respectively. Bland-Altman plots were used to illustrate the agreements between arm_OD and finger_OD during baseline sleep and under CPAP. We used *t* tests to determine whether these measurements significantly differed.

**Results:**

In total, 534 obstructive apneas and 2185 hypopneas were recorded. Of the 2185 hypopneas, 668 (30.57%) were collected during baseline sleep and 1517 (69.43%), during CPAP sleep. The mean difference between finger_OD and arm_OD was 2.86% (95% CI 2.67%-3.06%, t_667_=28.28; *P*<.001; 95% limits of agreement [LoA] –2.27%, 8.00%) during baseline sleep and 1.83% (95% CI 1.72%-1.94%, t_1516_=31.99; *P*<.001; 95% LoA –2.54%, 6.19%) during CPAP. Using the standard criterion of 3% saturation drop, arm_OD only recognized 16.32% (109/668) and 14.90% (226/1517) of hypopneas at baseline and during CPAP, respectively.

**Conclusions:**

arm_OD is 2% to 3% lower than standard finger_OD in sleep hypopnea, probably because the measured arm_OD originates physiologically from arterioles, venules, and capillaries; thus, the venous blood adversely affects its value. Our findings demonstrate that the standard criterion of ≥3% OD drop at the arm or wrist is not suitable to define hypopnea because it could provide large false-negative results in diagnosing OSA and assessing CPAP treatment effectiveness.

## Introduction

Monitoring health using wearables, such as smartwatches and armbands, is becoming a new lifestyle [1-4]. Hundreds of millions of smartwatches and armbands are being used daily, and the number is still sharply increasing. Sleep monitoring is one of the most popular functions of such wearables [2,5-7], because sleep is a critical determinant of an individual’s health and well-being. Obstructive sleep apnea (OSA) is the most prevalent respiratory sleep disorder occurring in 9% to 38% of the general population [8], and it is a high-risk factor for cardio- and cerebrovascular diseases [9,10]. Nevertheless, about 90% of suspected patients with OSA remain undiagnosed [11] due to the lack of sleep laboratories or specialists and the high cost associated with an in-lab polysomnography (PSG) diagnosis (ie, the gold-standard for sleep disorder diagnosis [12]), thus leading to decreased quality of life and increased health care burden in the aging society. Relatively simple and less-expensive diagnostic methods such as portable home respiratory polygraphy have been developed [13,14]. However, its signal quality is compromised, with failure rates ranging from 3% to 18% [14], mostly due to incorrect handling of the device or sensors by the people performing the test (ie, the patients themselves) [15]. Home respiratory polygraphy also has the risk of false diagnosis because it cannot measure sleep/wake, so patients may be awake during the night. The cost of such diagnostic methods is still relatively high in many low- and middle-income countries, limiting their broad application in the general population. Therefore, using low-cost and easy-to-use wearable devices, such as smart finger rings, smartwatches, or armbands, for at-home diagnosis of OSA and to assess treatment effectiveness would substantially contribute to public health worldwide [6,16].

However, using the aforementioned low-cost wearable devices to evaluate OSA is still not clinically viable because, currently, no product has been licensed or certified as a medical diagnostic device by the United States Food and Drug Administration (FDA) or CE marking. We hypothesize that one of the major limitations in measuring OSA with wearables is the detection of sleep hypopnea. Currently, most consumer-grade wearables can measure surrogate markers for breathing and heartbeats or heart rate variability [3,17-20]. Although sleep apneas consisting of a complete pause in breathing for ≥10 seconds are relatively easy to assess by analyzing breathing frequency [6,12,21-23], it is challenging to detect sleep hypopnea, which is defined as a ≥30% drop in airflow lasting ≥10 seconds accompanied by either an arousal or a ≥3% drop in peripheral capillary oxygen saturation (SpO_2_) measured at the fingertip [12]. Smart rings that can measure SpO_2_ at the fingertip (eg, Sleepon [24]) are likely to accurately quantify the drops in SpO_2_ because, essentially, they are similar to fingertip pulse oximetry. However, these devices have only a very tiny market share compared to other popular wearables such as smartwatches and armbands [25]. Whether the measures of oxygen desaturation (OD) at the fingertip and at the arm or wrist are physiologically identical in sleep hypopneas is essentially unknown. Some recent smartwatches (eg, Fitbit [26], Garmin [27], Huami [28], and Huawei [29]), armbands (eg, Humon [30,31], Moxy [32,33], PortaMon [34], and Biofourmis [35]), and prototypes [36,37] claim to measure SpO_2_ or muscle tissue oxygen saturation (StO_2_) at the arm or wrist. However, to the best of our knowledge, these devices have not been clinically validated for use in patients with OSA. We were able to find only one registered clinical validation study (Trial Registration: ClinicalTrials.gov NCT03775291) measuring OSA using a smartwatch, which was initiated by Fitbit in December 2018 [38]. However, the latest update of the study’s recruitment status as of November 2019 was still “active, not recruiting.” The study aimed to only compare PSG-assessed Apnea–Hypopnea Index (AHI) with the AHI derived from the smartwatch, rather than performing an event-by-event comparison of the apnea or hypopnea events measured by these two devices. Thus, even if we assume that Fitbit may have completed their validation work recently, their study still cannot answer the key question as to whether the hypopnea diagnostic standard criterion of ≥3% OD at the fingertip can be equally applied to the OD at the arm or wrist.

Therefore, we, for the first time, aimed to compare event-by-event OD at the fingertip (finger_OD) measured by the gold-standard in-lab PSG transmission photoplethysmography (T-PPG) with OD at the arm (arm_OD) measured by frequency-domain multidistance (FDMD) near-infrared spectroscopy (NIRS) in sleep hypopneas during naïve sleep and continuous positive airway pressure (CPAP) therapy. FDMD-NIRS is a well-validated [39-41] advanced, noninvasive optical technique that can quantify hemodynamic changes, including OD, in the measured tissues for long-term recordings such as all-night sleep with high temporal resolution [42,43]. Our results can conclusively demonstrate whether physiologically arm_OD measures can directly replace finger_OD to define sleep hypopnea. Thus, this study will have a broad appeal to the general public, sleep clinicians and scientists, health care insurance providers, and wearable technology developers who are aiming to measure OSA at-home by using wearable devices.

## Methods

### Patients

All patients underwent video-PSG measurement for diagnosis in our sleep laboratory. The following day, those patients who were diagnosed with OSA and recommended to use CPAP therapy by clinicians were recruited and gave their written informed consent for participation in the study. Patients with unstable coronary or cerebral artery disease, severe arterial hyper- or hypotension, respiratory diseases, or a history of a sleep-related accident were excluded. Finally, 30 newly diagnosed patients with OSA (mean age 54.2, SD 13.8 years, IQR 42-65 years; male: n=27; mean BMI 35.9, SD 7.5 kg/m^2^, IQR 31.8-42.0 kg/m^2^; mean AHI 53.4, SD 24.7 per hour, IQR 32-71 per hour) participated in this study. This study was approved by the local ethical commission of Northwest Switzerland, and it was in compliance with the Declaration of Helsinki.

### Protocol

The patients underwent incremental stepwise CPAP (AirSense 10, ResMed) titration combined with video-PSG and FDMD-NIRS recordings in one nocturnal sleep episode. This sleep episode consisted of 1 hour of baseline sleep without CPAP, followed by incremental stepwise titration of 1 cmH_2_O pressure per hour starting from 5-8 cmH_2_O depending on the individuals. The 1-h baseline sleep allowed us to compare arm_OD with finger_OD during natural sleep in patients with OSA. We included the CPAP titration protocol because (1) the stepwise CPAP titration protocol can increase the number of hypopneas for data analysis in our patients, since low pressures cannot fully open the airways to restore the apneas but instead cause hypopneas and (2) auto-CPAP that can automatically adjust the pressures within a given range (ie, automatic titration), and CPAP with fixed pressures are the most efficient and popular therapy for OSA [44]. Thus, the comparison between arm_OD and finger_OD in hypopneas under various CPAP pressures could allow us to test the feasibility of monitoring treatment efficacy by measuring arm_OD.

### Video-PSG

Video-PSG (Embla RemLogic, Natus Medical Incorporated) is a comprehensive recording of physiological signals during sleep, including electroencephalography at electrode locations of C3, C4, O1, O2, F3, and F4 according to 10 to 20 system, eye movements (electrooculogram), muscle activation (electromyogram), electrocardiogram, breathing functions, heart rate, fingertip SpO_2_ (left index fingertip in this study), and movement during sleep. Two experienced sleep technologists independently scored the sleep stages, respiratory and limb movement events, and motion artifacts in 30-second epochs according to the 2017 American Academy of Sleep Medicine manual [12]. Sleep hypopneas were defined as an at least 30% drop in airflow lasting for at least 10 seconds with either an arousal or >3% drop in SpO_2_. The discrepancy between these two technologists was resolved by discussion or recommendation by an experienced neurophysiologist. The hypopneas were excluded from analysis if their SpO_2_ desaturations were larger than 15% (n=31) to exclude outliers and potentially unreliable measurements caused by instrument errors.

### FDMD-NIRS Measurements

In this study, FDMD-NIRS (Imagent, ISS) measurements were conducted over the middle of the left biceps muscle. Imagent is currently the only commercial benchtop FDMD-NIRS device [40,42,45] and has been CE-approved for research. Its light emitters, four laser diodes at 690-nm wavelength and four laser diodes at 830-nm wavelength, are coupled into four light sources and are high frequency modulated at 110 MHz. The light can penetrate the measured tissues with a depth of several centimeters when the four light sources are aligned and placed at 2, 2.5, 3, and 3.5 cm away from an optical fiber bundle connected to the photomultiplier tube detector. 

**Figure 1:**
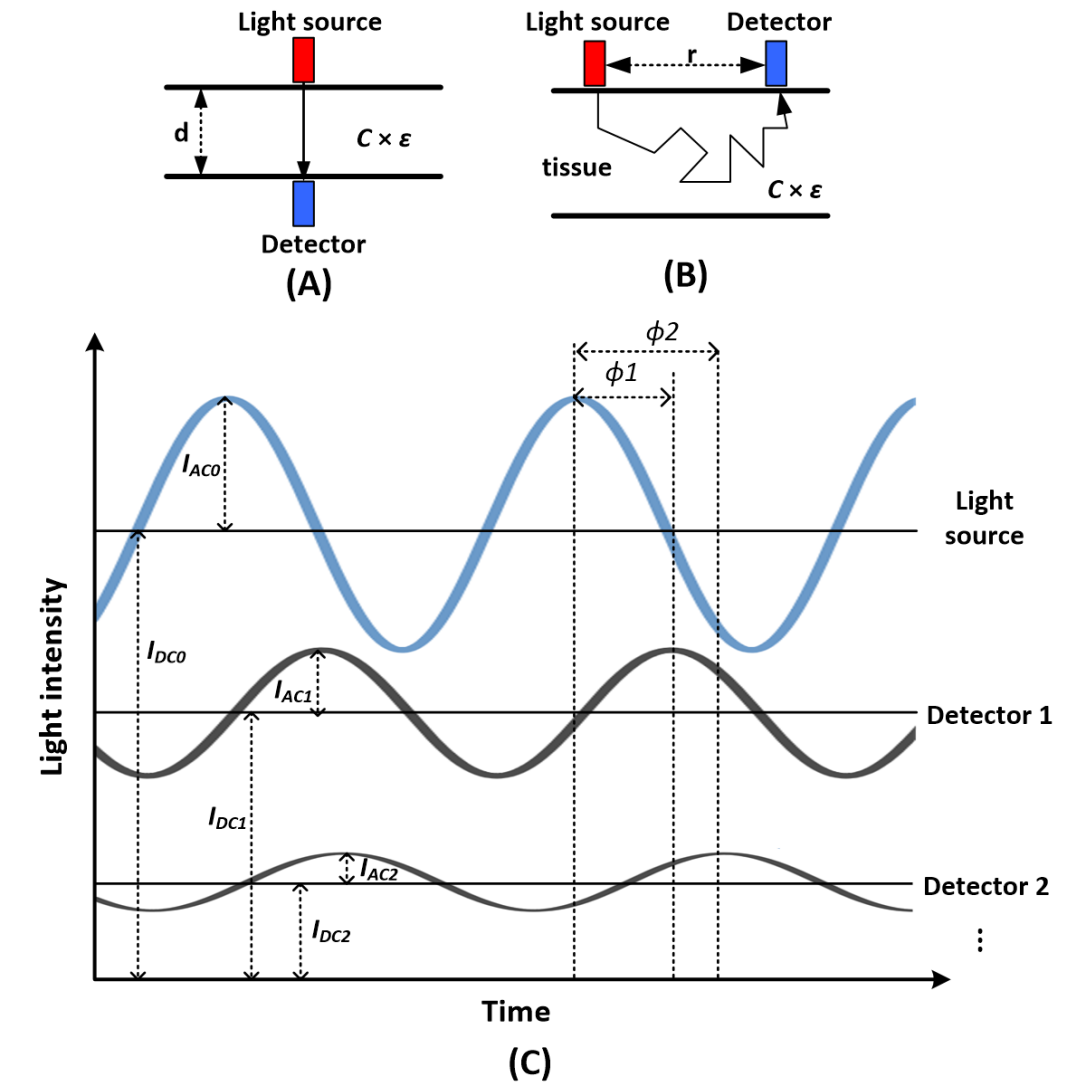
Beer–Lambert law and frequency-domain multidistance near-infrared spectroscopy (FDMD-NIRS) measurement. (A) The original Beer–lambert law describing the light propagation in a nonscattering absorbing media. The attenuation of light intensity in this absorbing media is proportional to the concentration C multiplied by the constant extinction coefficient ε for the particular absorber and the length d of the absorbing media. (B) The travelling of light in biological tissue (ie, highly scattering media). Light travels a longer pathway in the tissue than the light source-detector distance r due to scattering. (C) Basic principle of FDMD-NIRS measurement. The blue sine wave represents the high-frequency modulated light source. *I_DC0_* and *I_AC0_* are its light intensity and modulation amplitude, respectively. The two black sine waves are the output light detected after passing the measured tissues. They are detected by detectors 1 and 2 placed at different distances away from the light source. The light intensities and modulation amplitudes of the two black sine waves are smaller than those of the light source, and their phases are delayed because of the absorption and scattering in the tissues. Detector 1 is closer to the light source than detector 2. Therefore, the light intensity *I_DC1_* and modulation amplitude *I_AC1_* detected at detector 1 are larger than the light intensity *I_DC2_* and modulation amplitude *I_AC2_* detected at detector 2. The phase delay φ1 at detector 1 is smaller than the phase delay φ2 at detector 2 because the light reaching detector 2 has travelled a longer distance in the tissue. Similarly, the light intensity and modulation amplitude will be further decreased, and the phase delay will be further increased, when the light reaches the other detectors placed farther away than detector 2.

The most common commercially available NIRS devices, including wearable NIRS devices (eg, Humon [30,31], Moxy [32,33], and PortaMon [34]), are continuous-wave NIRS (CW-NIRS), which measure the hemodynamic changes in human tissues based on the modified Beer–Lambert law (MBLL) [46-48]. As illustrated in Figure 1A, in the original Beer–Lambert law, the light extinction is proportional to the concentration *C* multiplied by the constant extinction coefficient ε for the particular absorber and the length *d* of the absorbing media when light passes through a nonscattering but absorbing media [49]. *C* × ε is also called the absorption coefficient µ_a_ of the absorbing media. However, biological tissues are highly scattering media, and scattering will increase the path-length of light, thus increasing the probability of both light absorption and loss of light. Scattering also makes it possible that some light can go out of the tissue from the same side of the light source (ie, backscattering light), as shown in Figure 1B. Thus, reflectance photoplethysmography (R-PPG) pulse oximeter [50-52] and the NIRS device in which the light source and detector are placed on the same side of the measured tissues were developed based on MBLL. In MBLL, the light propagation due to scattering is taken into account by introducing the differential path-length factor (DPF). The real path-length of light in the tissue is then calculated as DPF multiplied by the source-detector distance *r*. DPF varies between different biological tissues and different individuals, and it also depends on other factors such as light wavelength and age and gender of the individual [48,53]. CW-NIRS devices use fixed value of DPF in the range of 3 to 6 for different light wavelengths [53]. They can only estimate the relative changes in the main absorbing chromophores, that is, oxygenated hemoglobin (HbO_2_) and deoxygenated hemoglobin (HHb) in the measured biological tissues. R-PPG and T-PPG pulse oximeters usually use two near-infrared wavelengths that are mainly absorbed by HbO_2_ and HHb respectively. The influences of scattering on light attenuation are approximately assumed to be cancelled out in the calculation of SpO_2_ that is derived from the ratio of the light intensities of the two wavelengths, ie, assuming the path-lengths of the two wavelengths are identical in the tissues [50-52]. However, this assumption is actually not valid because DPF varies between different wavelengths. Experimental calibrations against in vitro measurement of arterial oxygen saturation (SaO_2_) in extracted arterial blood (ie, invasive co-oximetry) thus must be performed in commercial pulse oximeters during their research and development period to correct measurement errors [50-52]. Several calibration-free methods were also proposed, including calculating the real light path-length and absorption by using the frequency-domain NIRS algorithm [52,54-57].

As illustrated in Figure 1C, in FDMD-NIRS, the light emitted from light source can be detected by detectors placed at different distances away from the source. The light intensity (*I_DC_*) and modulation amplitude (*I_AC_*) of the detected light decrease, and a phase delay (φ) occurs between the detected light and the source light due to absorption and scattering. The detected *I_DC_* and *I_AC_* are smaller but theφ is larger at the detector further away from the light source. The *I_DC_*, *I_AC_* and φ from different light source-detector distances vary linearly [42,45]. Therefore, to submit the measured *I_DC_*, *I_AC_* and φ to linear regression, we can obtain the following equations derived from the photon diffusion equation in a semi-infinite geometry [40,45,58-60]:

*ln (r^2^I_AC_) = rS_AC_ + C_AC_*** (1)**

*ln (r^2^I_DC_) = rS_DC_ + C_DC_*** (2)**

φ = *rS*_φ_ + *C*_φ_** (3)**

Where *r* is the known source-detector distance, *S_AC_*, *S_DC_* and *S*_φ_ are the slopes and *C_AC_*, *C_DC_* and *C*_φ_ are the intercepts. The linearity of the equation is monitored by the *R^2^* of the fitted linear regression. By combing any two of these three slopes (eg, we chose *S_AC_* and *S*_φ_ in the following equations), we can calculate the absorption coefficient µ_a_ and the reduced scattering coefficient µ_S_^’^ of the measured tissue [40,45,58-60]:

*µ_a_* = ω/*2v* × (*S*_φ_ / *S_AC_ −S_AC_* / *S*_φ_)** (4)**

*µ_S_^’^* = (*S_AC_^2^ − S*_φ_*^2^*) / *3µ_a_−µ_a_*** (5)**

where ω/*2π* is the modulation frequency and *v* is the velocity of light in the tissue. FDMD-NIRS uses two wavelengths. The µ_a_ and µ_S_^’^ of both these wavelengths can be calculated individually by using the same equations (4) and (5). Equation (4) gives us the absorption coefficient of the measured tissues calculated by taking the influence of scattering into account. It is equal to *C* × ε as mentioned above. In NIRS, the main contributions to absorptions in tissues are HbO_2_ and HHb, so we have the following equation:

*µ_a_^λ^* = *ε_HHb_^λ^ C_HHb_* + *ε_HbO2_^λ^ C_HbO2_*** (6)**

where µ_a_^λ^ is the absorption coefficient of the measured tissue at wavelength λ. ε_HHb_^λ^ and ε_HbO2_^λ^ are the known extinction coefficients at wavelength λ for HHb and HbO_2_, respectively. *C_HHb_* and *C_HbO2_* are the concentrations of HHb and HbO_2_, respectively. Using two wavelengths λ1 and λ2, we can then calculate *C_HHb_* and *C_HbO2_* with the following equations:

*C_HbO2_* = *(μ_a_^λ1^ ε_HHb_^λ2^* − *μ_a_^λ2^ ε_HHb_^λ1^)* / *(ε_HbO2_^λ1^ ε_HHb_^λ2^* − *ε_HbO2_^λ2^ ε_HHb_^λ^)*** (7)**

*C_HHb_* = *(μ_a_^λ2^ ε_HbO2_^λ1^* − *μ_a_^λ1^ ε_HbO2_^λ2^)* / *(ε_HbO2_^λ1^ ε_HHb_^λ2^* − *ε_HbO2_^λ2^ ε_HHb_^λ1^)*** (8)**

Therefore, Sto_2_ can be further derived as:

*StO_2_* = *100* × *C_HbO2_* / *(C_HbO2_* + *C_HHb_)*** (9)**

The FDMD-NIRS algorithm can calculate the absolute values of HbO_2_, HHb, and StO_2_ in the measured tissue and it is superior to the simple CW-NIRS algorithm, because of its sophisticated mathematical frameworks calculating µ_a_ and µ_S_^’^ that can best estimate the real light propagation distance in the measured tissues based on the diffusion equation in complex geometries. The robustness, precision, and accuracy of measuring HbO_2_, HHb, and StO_2_ of the Imagent system used in this study have been well validated in different physical blood-lipid models [45,58,61] and in vivo studies [59,62-64]. It has been used as a gold-standard reference measurement of StO_2_ for validations or calibrations of wearable CW-NIRS armbands [31] and portable CW-NIRS oximeters [61,65] including those that have received FDA clearance [66,67].

In this study, the sampling rate of FDMD-NIRS recording was 5.2 Hz. The reliability and accuracy of FDMD-NIRS measurements depend on the linearity of the measured optical signals on distances, because µ_a_ and µ_S_^’^ are derived from the slopes of equations (1-3). The linear dependence *R^2^* of the modulated light amplitude and phase shift over the measured distances should be highly close to 1 in each light wavelength. Thus, before the start of the recording, our Imagent system was calibrated on an optical phantom block with known µ_a_ and µ_S_^’^, that is, the light intensity of each light source was adjusted so that *R^2^* was equal to 1 and the measured µ_a_ and µ_S_^’^ of the optical phantom block were equal to their known values. This calibration step can exclude the uncertainty of our measurements due to machine errors such as light source and detector errors. The raw optical data were discarded if the *R^2^* was smaller than 0.95 in either modulated light amplitude or phase shift in any wavelength to exclude poor-quality data arising from improper probe-skin contact and shunted light reaching the detector without travelling through the tissue [68,69]. The NIRS data were then subjected to a low-pass (<0.08 Hz), zero-phase filter designed using Hanning window to remove the physiological noises, including heart rate, respiratory noise, and spontaneous slow hemodynamic oscillations [70,71]. The filtered data were smoothed with moving average smooth method (robust locally weighted scatter plot smoothing [70,72]).

### Statistical Analysis

Bland-Altman plots were used to illustrate the agreements between arm_OD and finger_OD during baseline sleep and under CPAP, respectively. We used *t* test to determine whether the differences between arm_OD and finger_OD were significantly different from 0. In order to check whether arm_OD could replace finger_OD in subgroups of hypopneas (ie, hypopneas with different degrees of desaturations), we used Spearman correlation to evaluate the relationship between arm_OD and finger_OD in different subgroups defined by finger_OD greater than or equal to specific cut-offs. The cut-offs ranged from 2% to 8%. *P* values <.05 indicated statistical significance for both analyses. Pre-processing of FDMD-NIRS signals was carried out in MATLAB (MathWorks, Inc.). All statistical analyses were performed using R statistical software (version 3.2.4; R Foundation for Statistical Computing).

## Results

**Figure 2:**
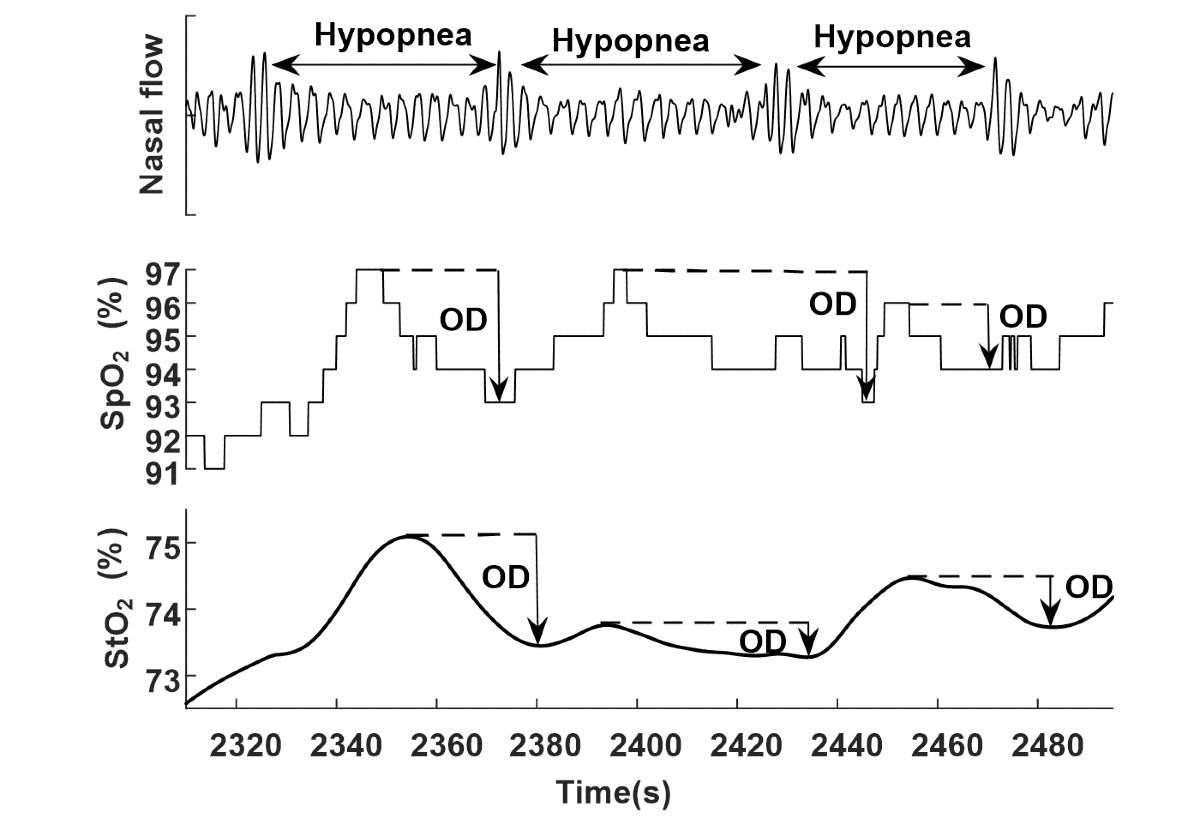
Typical oxygen desaturation (OD) at fingertip (finger_OD) and at arm (arm_OD) during hypopneas. Arrows indicate the degree of OD. SpO_2_ is measured at the fingertip by polysomnography transmission photoplethysmography, and StO_2_ is measured at the biceps muscle by frequency-domain multidistance near-infrared spectroscopy. SpO_2_: peripheral capillary oxygen saturation; StO_2_: peripheral tissue oxygen saturation.

**Figure 3:**
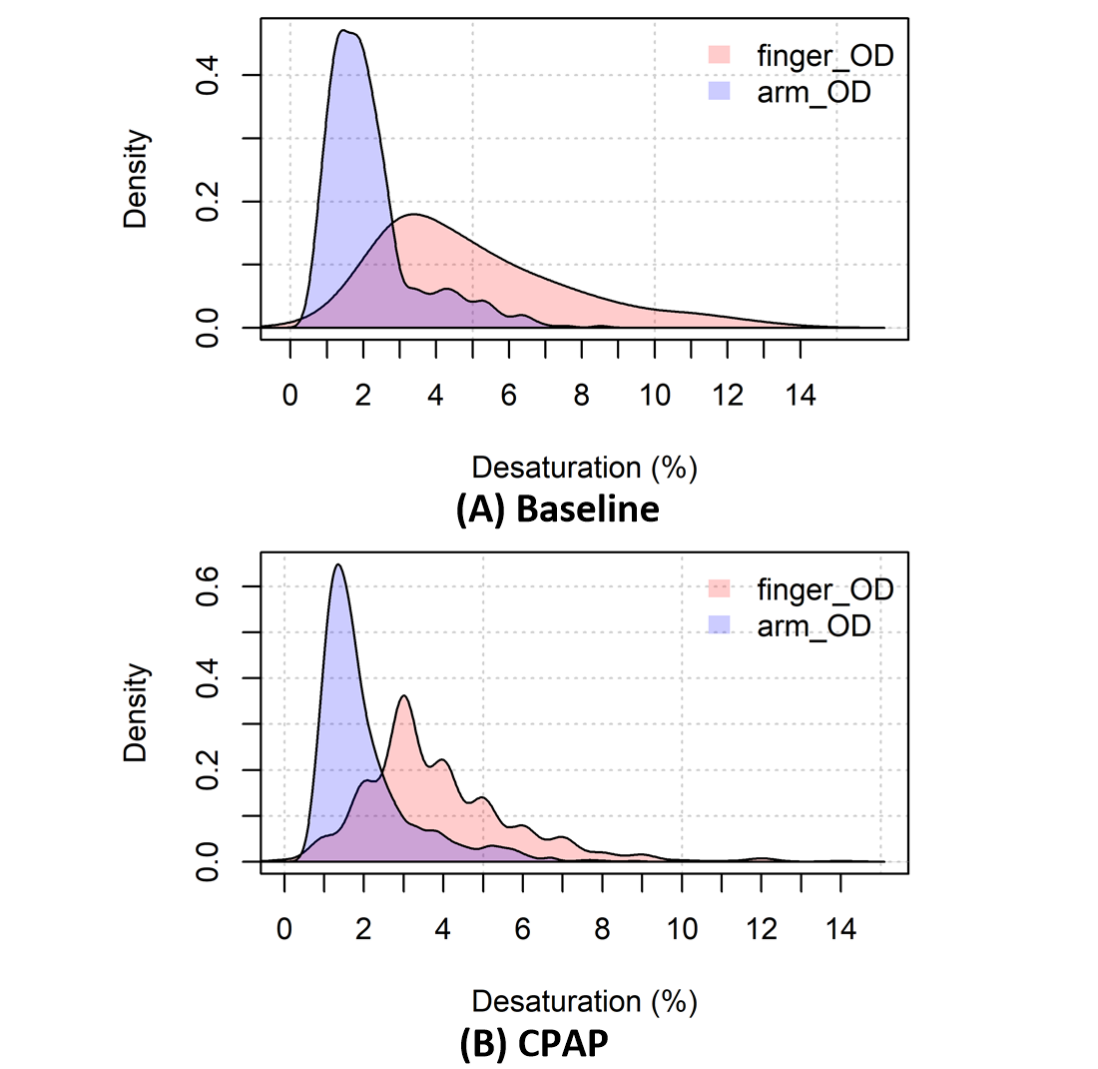
Distributions of oxygen desaturation at fingertip (finger_OD) and at arm (arm_OD) at (A) baseline (n=668) and (B) under continuous positive airway pressure (CPAP: n=1517).

In total, 2185 hypopneas (median 67, IQR 41-82) were analyzed, including 668 (median 16, IQR 11-35) recorded during baseline sleep and 1517 (median 41, IQR 25-64) recorded during CPAP. Figure 2 illustrates the typical changes that occurred in arm_OD and finger_OD in hypopnea events. Indeed, sleep hypopneas cause OD in arm StO_2_. The absolute (mean) values of StO_2_ are 68.8% (SD 6.5%) at baseline before the start of hypopneas. The distributions of finger_OD and arm_OD (Figure 3) suggest larger OD in the finger than in the arm both at the baseline and during CPAP, as the peaks of the distributions of arm_OD are smaller than those of finger_OD.

**Figure 4:**
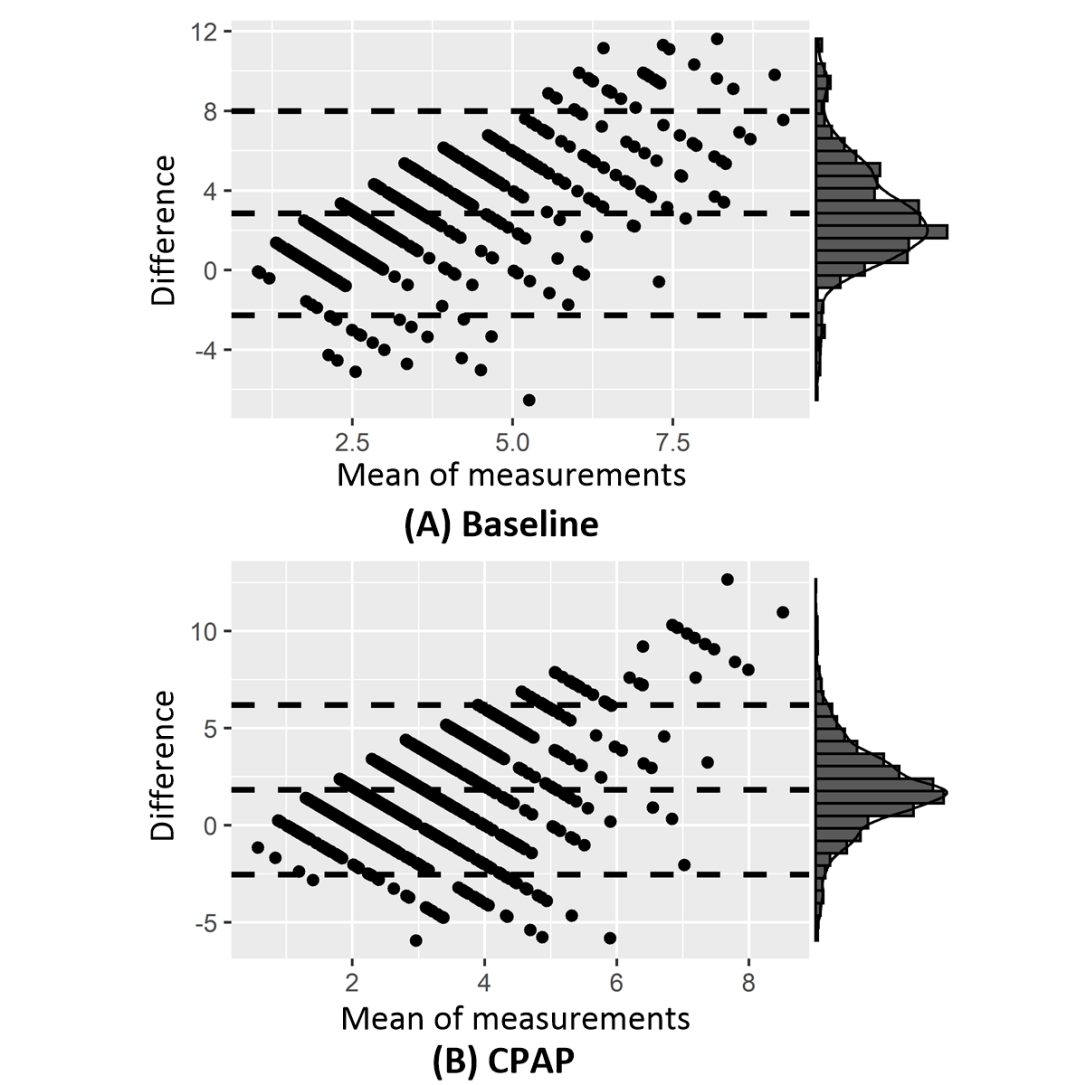
Bland-Altman plots of oxygen desaturation at fingertip (finger_OD) and at arm (arm_OD) at (A) baseline (n=668) and (B) under continuous positive airway pressure (CPAP: n=1517). The x-axes show the mean between the two measures, whereas the y-axes represent the differences (ie, finger_OD – arm_OD). The horizontal dotted lines indicate the mean difference and the 95% limits of agreement between the measures, ie, mean difference ± 1.96 × SD. The distribution of the mean difference is shown at the right margin of the plot, which is a normal distribution.

Bland-Altman plots (Figure 4) show that the mean difference between finger_OD and arm_OD is 2.86% (95% CI 2.67%-3.06%, *t_667_*=28.28; *P*<.001) during baseline sleep and 1.83% (95% CI 1.72%-1.94%, t_1516_=31.99; *P*<.001) under continuous positive airway pressure (CPAP) sleep, with broad 95% limits of agreement (LoA) as [-2.27%, 8.00%] and [-2.54%, 6.19%], respectively. Using the criterion of arm_OD ≥3%, we can only define 16.32% (109/668) and 14.90% (226/1517) of hypopneas at baseline and during CPAP sleep, respectively.

**Figure 5:**
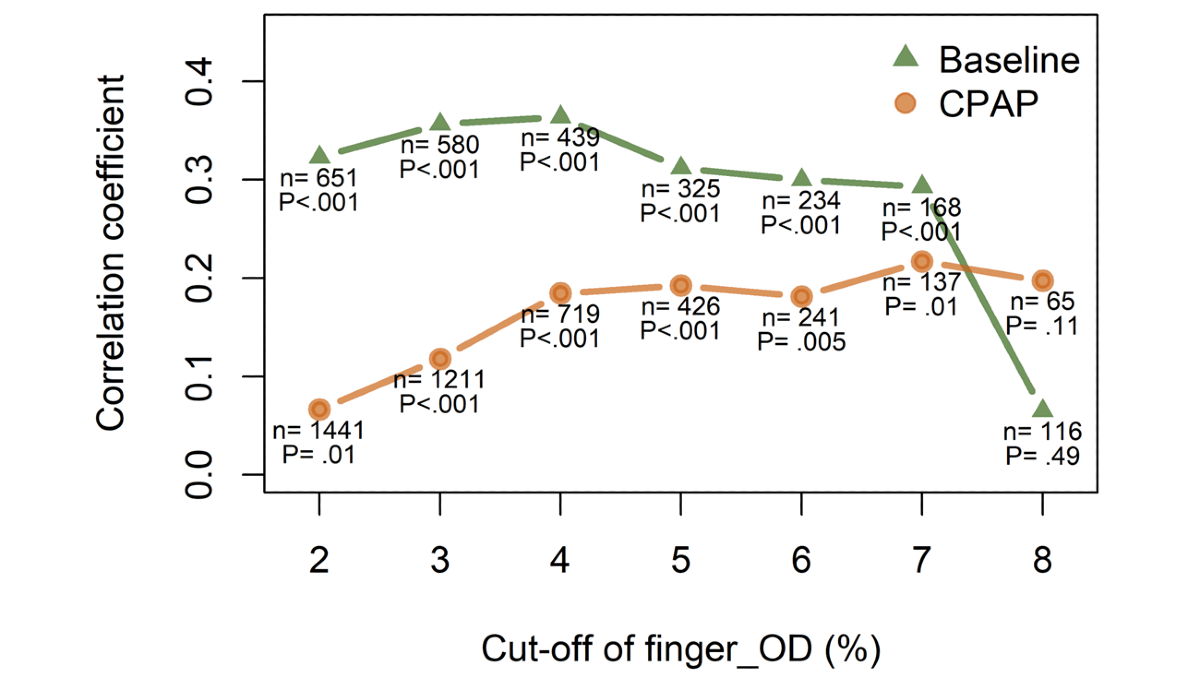
The correlations between oxygen desaturation at fingertip (finger_OD) and at arm (arm_OD) in the hypopnea events wherein finger_OD ≥ cut-off. X-axis shows the cut-off of finger_OD (from 2% to 8%). Y-axis depicts nonparametric Spearman’s correlation coefficient between finger_OD and arm_OD. The number of events used for correlation analysis and the *P* value are shown in the figure. The correlation analyses are performed for both baseline and CPAP sleep. For example, the green triangle at X=6, Y=0.3 means that during naïve baseline sleep there are 234 hypopneas associated with at least 6% finger_OD, and in these events, arm_OD weakly correlates to finger_OD with Spearman’s correlation coefficient equal to 0.3 and *P*<.001.

To test whether arm_OD could replace finger_OD in subgroups of hypopneas such as those causing severe OD, we correlate arm_OD and finger_OD in different subgroups with finger_OD ≥ specific cut-offs (from 2% to 8%). Again, we could only observe weak correlations (ie, correlation coefficients < 0.4) between them at baseline and under CPAP (Figure 5).

Since the baseline values of StO_2_ (mean 68.8% SD 6.5%) are obviously much smaller than those of SpO_2_ (usually above 90%), it is possible that the relative change rather than the absolute (raw) value of arm_OD has better agreement with the finger_OD. We therefore normalize the arm_OD to its baseline. The mean difference between finger_OD and normalized arm_OD is 1.68% (95% CI 1.46-1.90%, *t_667_*=14.99; *P*<.001) during baseline sleep and 0.88% (95% CI: 0.75-1.01%, *t_1516_*=12.91; *P*<.001) during CPAP sleep, respectively. The mean differences are smaller than those between absolute arm_OD and finger_OD. However, the 95% LoA are still equally broad, which are [-3.99%, 7.34%] during baseline sleep and [-4.32%, 6.08%] during CPAP sleep respectively, suggesting poor agreement. Only 41.6% (278/668) of hypopneas at baseline and 31.7% (481/1517) during CPAP sleep can be defined using the criterion of normalized arm_OD ≥3%, suggesting that the normalization still has poor sensitivity in detecting hypopneas.

## Discussion

### Principal Findings

In this study, we compare, for the first time, event-by-event finger_OD to arm_OD in sleep hypopneas during naïve sleep and CPAP therapy sleep. We choose the gold-standard reference methods for both arm_OD and finger_OD—advanced FDMD-NIRS for arm_OD and PSG fingertip transmission photoplethysmography for finger-OD. Arm_OD is 2% to 3% smaller than finger_OD, probably because finger_OD is caused only by arterioles, whereas arm_OD is physiologically determined by mixed sources of arterioles, venules, and capillaries [42,73,74]. The lower value of arm_OD is thus most likely due to the contribution of the venous blood pool. The significant difference between finger_OD and arm_OD and their broad LoA (Figure 4) suggest that arm_OD cannot directly replace finger_OD to define hypopneas. If the standard criterion of ≥3% drop is applied, it will cause a high rate of false-negative results in diagnosing OSA and in assessing the efficacy of CPAP treatment. The poor agreement between finger_OD and arm_OD and the low sensitivity of arm_OD in measuring hypopneas cannot essentially be improved even after normalization, in which raw arm_OD is normalized to its baseline before the start of hypopnea. The poor correlations (Figure 5) between finger_OD and arm_OD across hypopnea severity further suggest that the arm_OD cannot be used to define hypopneas, that is, correlations remain low even in severe hypopneas that are associated with much larger finger_ODs.

The armband and smartwatch are two of the most popular wearable technologies. Our results have direct implications for wearable armbands and portable oximeters using NIRS techniques to measure OSA, as we discuss below.

First, the FDMD-NIRS system used in our study is well recognized as the most robust and reliable reference NIRS technique [31,42,61]. Arm StO_2_ measured by our device should be identical to that measured by consumer-grade wearable NIRS armbands (eg, Humon [30,31], Moxy [32,33], and PortaMon [34]). It should also be equal to that measured by FDA-cleared medical-grade portable NIRS oximeters, such as INVOS 5100C (Medtronic) [66], FORE-SIGHT (CAS Medical Systems) [67], SenSmart (Nonin Medical Inc) [75], Hutchinson InSpectra (Hutchinson Technology Inc) [76], and ViOptix ODISsey (ViOptix Inc) [77]. Our results suggest that the absolute or relative (ie, normalized to baseline) OD at arm muscle measured by these aforementioned devices cannot be directly used to define sleep hypopnea.

Second, the NIRS StO_2_ is the proportion of HbO_2_ in arterial, capillary, and venous compartments of the measured tissue. It can be expressed by its two major compounds as StO_2_ = (*a* × SaO_2_) + (*b* × SvO_2_), where SaO_2_ and SvO_2_ are the arterial and venous oxygen saturation [68,73]. Fingertip SpO_2_ is the best noninvasive estimate of SaO_2_ [50,52]. The ratio of the coefficients a/b is called the arterio-venous ratio (AVR). The AVR of different tissues (eg, brain and muscles) and different populations (eg, healthy people and patients, adults, and children) has been determined by invasive measurements under different conditions but not during sleep [73,78-88]. Commercially available NIRS oximeters, including the aforementioned FDA-certificated medical devices take the fixed AVR value as either 0.25/0.75 or 0.30/0.70 but have not validated it in OSA [78-88]. Our results of poor correlations between arm_OD and finger_OD (Figure 5) indicate that such an uncritical AVR adoption is not justified. According to the mathematical model of fixed relationship between StO_2_ and SaO_2_, we expect a strong correlation between StO_2_ and SpO_2_ that we could not confirm using our data. These conflicting results suggest that the model is not valid for OSA. Therefore, developers of wearable or portable NIRS devices should first study the AVR in muscle tissues and validate it against invasive blood sample measurements before using their devices commercially for OSA diagnosis.

Our findings also indicate that appropriate arterial oxygen saturation measurement is not possible with armbands using the R-PPG method. As mentioned in the Methods section, R-PPG essentially has the same theoretical limitations as CW-NIRS, such that the scattering of light in the human tissues cannot be calculated. R-PPG and T-PPG pulse oximeters estimate SpO_2_ under the assumption that changes in blood volume only occur in arterial but not in the venous compartment. SpO_2_ is measured from the fingertip or the superficial forehead because these locations are well perfused by arteries [36,52,89]. It is not recommended to measure SpO_2_ by using R-PPG at the wrist or the arm, because of its low signal-to-noise ratio. Compared to the more precise fingertip T-PPG method, the low signal-to-noise ratio of R-PPG at the wrist or arm is about 10 times weaker due to various factors such as relative low blood perfusion and sensitivity to pressure and ambient light sources [51,89]. Additionally, the key assumption of constant venous blood volume is no longer valid at the wrist or arm [89,90]. Although recently, some smartwatches [26-29] and a few armbands [35-37] claimed that they can measure SpO_2_ at the arm or wrist by using R-PPG, they actually measure both arterial and venous blood [89,90] similar to NIRS. A main difference between NIRS and R-PPG is the measurement depth. Whether R-PPG measures the blood only in the skin or in both the skin and muscle depends on the distance between the light source and detector [91]. The detector can detect the light passing through deeper tissues at a larger separation distance. In vivo studies suggest that R-PPG can obtain its best signal-to-noise ratio at a separation distance of 3 to 6 mm [92]. Thus, a separation distance of several millimeters is used in the design of R-PPG pulse oximetry [36]. A recent study modeled the R-PPG light propagation in human skin [37]. The authors found that even at a separation distance of only 0.6 mm, many light rays reaching deeper into the muscle can still be received by the detector because of the random nature of light scattering [37]. Therefore, similar to NIRS armbands, armband devices using R-PPG capture the oxygen saturation in both skin and muscle. There are challenges in using both techniques to differentiate SaO_2_ and SvO_2_ desaturations from the measured StO_2_ desaturation to define sleep hypopneas.

We did not include a smartwatch in this study, although recently, leading smartwatch companies like Apple, Fitbit, Garmin, Huami, and Huawei have all added the function of measuring oxygen saturation in their products. This is because these commercially available products cannot or are unwilling to export their raw data for analyses, or their temporal resolutions are simply too low for an event-by-event comparison necessary for our study because usually these consumer-grade smartwatches upload their data to their cloud servers in minute resolution. Nevertheless, our results may have indirect implications for smartwatches. R-PPG smartwatch is different from the fingertip T-PPG in measuring SpO_2_. Lee et al [89] found that the raw light signals measured by wrist R-PPG and fingertip T-PPG change differently during breath-holding, indicating different SpO_2_ values are calculated by these two techniques. Abay et al [36] reported that wrist R-PPG results in lower SpO_2_ values than fingertip T-PPG at rest, and during venous occlusion, fingertip T-PPG SpO_2_ does not exhibit desaturation but wrist R-PPG SpO_2_ drops similarly as the simultaneously measured NIRS StO_2_ at the same arm. Their findings also indicate that although smartwatches measure the wrist and NIRS measures the arm muscle, the measured changes in oxygen saturation by these two techniques are likely to be the same. Thus, the venous blood influences in our NIRS StO_2_ measurements are also likely to be observed in smartwatch measurements.

### Conclusions

Our study warns consumers, health care insurance companies, and sleep clinicians and scientists to interpret the AHI provided by smartwatches and armbands with caution until those products are clinically and experimentally validated. An AHI >5/hour suggests the diagnosis of OSA [12]. Our results suggest that AHI is likely to be underestimated if using the criterion of arm_OD ≥3% to define hypopneas. Wearable technology developers who are validating their products can learn from this study and take into account the mismatch between the ODs measured by their products and by the gold-standard technique fingertip pulse oximetry. Developing new parameters (eg, estimated-oxygen-variation provided by Fitbit smartwatches [26]) or combining smartwatches with external fingertip T-PPG sensors [93] may be a more promising strategy to measure OSA. Nevertheless, validations of these new approaches are necessary before releasing them for clinical use. In addition, our finding of a weak correlation between finger_OD and arm_OD indicates that (1) prediction of finger_OD using arm_OD may be possible but will require development and implementation of sophisticated data-mining, such as machine learning algorithms [90], and (2) as previous studies have validated NIRS oximeters as medical devices, protocols that quantify the arterial and venous contributions to the arm_OD are needed. Arm_OD can then be calibrated to indicate the changes in the arterial ODs at the arm or wrist.

## Abbreviations

AHI: Apnea–Hypopnea Index
arm_OD: arm oxygen desaturation
AVR: arterio-venous ratio
CPAP: continuous positive airway pressure
CW-NIRS: continuous wave near-infrared spectroscopy
DPF: differential path-length factor
FDMD: frequency-domain multidistance
finger_OD: fingertip oxygen desaturation
HbO_2_: oxygenated hemoglobin
HHb: deoxygenated hemoglobin
LoA: limit of agreement
MBLL: modified Beer–Lambert law
NIRS: near-infrared spectroscopy
OD: oxygen desaturation
OSA: obstructive sleep apnea
PSG: polysomnography
R-PPG: reflectance photoplethysmography
SaO_2_: arterial oxygen saturation
SNR: signal-to-noise ratio
SpO_2_: peripheral capillary oxygen saturation
StO_2_: peripheral tissue oxygen saturation
SvO_2_: venous oxygen saturation
T-PPG: transmission photoplethysmography

## References

[1] Depner C, Cheng P, Devine J, Khosla S, de Zambotti Massimiliano, Robillard R, Vakulin A, Drummond Sean P A (2020). Wearable technologies for developing sleep and circadian biomarkers: a summary of workshop discussions. Sleep.

[2] Zhang Z, Cajochen C, Khatami R (2019). social jetlag and chronotypes in the chinese population: analysis of data recorded by wearable devices. J Med Internet Res.

[3] Perez MV, Mahaffey KW, Hedlin H, Rumsfeld JS, Garcia A, Ferris T, Balasubramanian V, Russo AM, Rajmane A, Cheung L, Hung G, Lee J, Kowey P, Talati N, Nag D, Gummidipundi SE, Beatty A, Hills MT, Desai S, Granger CB, Desai M, Turakhia MP (2019). Large-scale assessment of a smartwatch to identify atrial fibrillation. N Engl J Med.

[4] Wen D, Zhang X, Liu X, Lei J (2017). evaluating the consistency of current mainstream wearable devices in health monitoring: a comparison under free-living conditions. J Med Internet Res.

[5] Haghayegh S, Khoshnevis S, Smolensky MH, Diller KR, Castriotta RJ (2019). Accuracy of wristband fitbit models in assessing sleep: systematic review and meta-analysis. J Med Internet Res.

[6] Baron KG, Duffecy J, Berendsen MA, Cheung Mason I, Lattie EG, Manalo NC (2018). Feeling validated yet? a scoping review of the use of consumer-targeted wearable and mobile technology to measure and improve sleep. Sleep Med Rev.

[7] Reimer U, Emmenegger S, Maier E, Zhang Z, Khatami R (2017). Recognizing sleep stages with wearable sensors in everyday settings.

[8] Senaratna CV, Perret JL, Lodge CJ, Lowe AJ, Campbell BE, Matheson MC, Hamilton GS, Dharmage SC (2017). Prevalence of obstructive sleep apnea in the general population: a systematic review. Sleep Med Rev.

[9] Yaggi HK, Concato J, Kernan WN, Lichtman JH, Brass LM, Mohsenin V (2005). Obstructive sleep apnea as a risk factor for stroke and death. N Engl J Med.

[10] Somers VK (2005). Sleep--a new cardiovascular frontier. N Engl J Med.

[11] Young T, Evans L, Finn L, Palta M (1997). Estimation of the clinically diagnosed proportion of sleep apnea syndrome in middle-aged men and women. Sleep.

[12] Berry R, Brooks R, Gamaldo CE, Harding SM, Lloyd RM, Marcus CL, Vaughn BV (2017). The AASM Manual for the Scoring of Sleep and Associated Events: Rules, Terminology and Technical Specifications.

[13] Masa JF, Corral J, Pereira R, Duran-Cantolla J, Cabello M, Hernandez-Blasco L, Monasterio C, Alonso A, Chiner E, Rubio M, Garcia-Ledesma E, Cacelo L, Carpizo R, Sacristan L, Salord N, Carrera M, Sancho-Chust JN, Embid C, Vazquez-Polo F, Negrin MA, Montserrat JM (2011). Effectiveness of home respiratory polygraphy for the diagnosis of sleep apnoea and hypopnoea syndrome. Thorax.

[14] Collop NA, Anderson WM, Boehlecke B, Claman D, Goldberg R, Gottlieb DJ, Hudgel D, Sateia M, Schwab R, Portable Monitoring Task Force of the American Academy of Sleep Medicine (2007). Clinical guidelines for the use of unattended portable monitors in the diagnosis of obstructive sleep apnea in adult patients. Portable Monitoring Task Force of the American Academy of Sleep Medicine. J Clin Sleep Med.

[15] Tan H, Gozal D, Ramirez H, Bandla H, Kheirandish-Gozal L (2014). Overnight polysomnography versus respiratory polygraphy in the diagnosis of pediatric obstructive sleep apnea. Sleep.

[16] Penzel T, Schöbel C, Fietze I (2018). New technology to assess sleep apnea: wearables, smartphones, and accessories. F1000Res.

[17] Zhang H, Zhang J, Li H, Chen Y, Yang B, Guo Y, Chen Y (2019). validation of single centre pre-mobile atrial fibrillation apps for continuous monitoring of atrial fibrillation in a real-world setting: pilot cohort study. J Med Internet Res.

[18] Fan Y, Li Y, Li J, Cheng W, Shan Z, Wang Y, Guo Y (2019). Diagnostic performance of a smart device with photoplethysmography technology for atrial fibrillation detection: pilot study (Pre-mAFA II Registry). JMIR Mhealth Uhealth.

[19] Chen E, Jiang J, Su R, Gao M, Zhu S, Zhou J, Huo Y (2020). A new smart wristband equipped with an artificial intelligence algorithm to detect atrial fibrillation. Heart Rhythm.

[20] Zhang Z, Henzmann S, Hügli G, Qi M, Chen W, Lu C, Khatami R (2018). Validation of wearable sleep monitoring device based on cardiopulmonary coupling and accelerometer with comparison to polysomnography in adults.

[21] Papini GB, Fonseca P, Gilst MMV, Bergmans JW, Vullings R, Overeem S (2020). Respiratory activity extracted from wrist-worn reflective photoplethysmography in a sleep-disordered population. Physiol Meas.

[22] Taffoni F, Rivera D, La Camera A, Nicolò Andrea, Velasco JR, Massaroni C (2018). A wearable system for real-time continuous monitoring of physical activity. J Healthc Eng.

[23] Aliverti A (2017). Wearable technology: role in respiratory health and disease. Breathe (Sheff).

[24] Go2sleep SE.

[25] Seneviratne S, Hu Y, Nguyen T, Lan G, Khalifa S, Thilakarathna K, Hassan M, Seneviratne A (2017). A survey of wearable devices and challenges. IEEE Commun Surv Tutorials.

[26] How do I track my estimated oxygen variation in the Fitbit app?.

[27] Top FAQs for the Pulse Ox Feature on Garmin Watches.

[28] Amazfit X Bow to the Future.

[29] HUAWEI Watch 3. Huawei Global.

[30] Humon Muscle Oxygen Sensor.

[31] Farzam P, Starkweather Z, Franceschini MA (2018). Validation of a novel wearable, wireless technology to estimate oxygen levels and lactate threshold power in the exercising muscle. Physiol Rep.

[32] The Science Behind Moxy.

[33] Feldmann A, Schmitz R, Erlacher D (2019). Near-infrared spectroscopy-derived muscle oxygen saturation on a 0% to 100% scale: reliability and validity of the Moxy Monitor. J Biomed Opt.

[34] PortaMon.

[35] What does the Everion measure?.

[36] Abay TY, Kyriacou PA (2015). Reflectance photoplethysmography as noninvasive monitoring of tissue blood perfusion. IEEE Trans Biomed Eng.

[37] Lee H, Kim E, Lee Y, Kim H, Lee J, Kim M, Yoo H, Yoo S (2018). Toward all-day wearable health monitoring: an ultralow-power, reflective organic pulse oximetry sensing patch. Sci Adv.

[38] Validation of Software for Assessment of Sleep Apnea From Data Acquired by a Wearable Smartwatch.

[39] Toronov VY, Zhang X, Webb AG (2007). A spatial and temporal comparison of hemodynamic signals measured using optical and functional magnetic resonance imaging during activation in the human primary visual cortex. NeuroImage.

[40] Toronov V, Webb A, Choi JH, Wolf M, Safonova L, Wolf U, Gratton E (2001). Study of local cerebral hemodynamics by frequency-domain near-infrared spectroscopy and correlation with simultaneously acquired functional magnetic resonance imaging. Opt Express.

[41] Gatto R, Hoffman W, Mueller M, Flores A, Valyi-Nagy T, Charbel FT (2006). Frequency domain near-infrared spectroscopy technique in the assessment of brain oxygenation: a validation study in live subjects and cadavers. J Neurosci Methods.

[42] Fantini S, Sassaroli A (2020). Frequency-domain techniques for cerebral and functional near-infrared spectroscopy. Front Neurosci.

[43] Mensen A, Zhang Z, Qi M, Khatami R (2016). The occurrence of individual slow waves in sleep is predicted by heart rate. Sci Rep.

[44] Berry R, Parish J, Hartse Kristyna M (2002). The use of auto-titrating continuous positive airway pressure for treatment of adult obstructive sleep apnea. An American Academy of Sleep Medicine review. Sleep.

[45] Fantini S, Franceschini MA, Maier J, Walker S, Barbieri B, Gratton E (1995). Frequency-domain multichannel optical detector for noninvasive tissue spectroscopy and oximetry. Optical Engineering.

[46] Villringer A, Chance B (1997). Non-invasive optical spectroscopy and imaging of human brain function. Trends Neurosci.

[47] Delpy DT, Cope M, van der Zee P, Arridge S, Wray S, Wyatt J (1988). Estimation of optical pathlength through tissue from direct time of flight measurement. Phys Med Biol.

[48] Scholkmann F, Kleiser S, Metz AJ, Zimmermann R, Mata Pavia J, Wolf U, Wolf M (2014). A review on continuous wave functional near-infrared spectroscopy and imaging instrumentation and methodology. NeuroImage.

[49] Beer (1852). Bestimmung der Absorption des rothen Lichts in farbigen Flüssigkeiten. Article in German. Annalen der Physik.

[50] Chan ED, Chan MM, Chan MM (2013). Pulse oximetry: Understanding its basic principles facilitates appreciation of its limitations. Respiratory Medicine.

[51] König V, Huch R, Huch A (1998). Reflectance pulse oximetry--principles and obstetric application in the Zurich system. J Clin Monit Comput.

[52] Nitzan M, Romem A, Koppel R (2014). Pulse oximetry: fundamentals and technology update. Med Devices (Auckl).

[53] Scholkmann F, Wolf M (2013). General equation for the differential pathlength factor of the frontal human head depending on wavelength and age. J Biomed Opt.

[54] Franceschini MA, Gratton E, Fantini S (1999). Noninvasive optical method of measuring tissue and arterial saturation: an application to absolute pulse oximetry of the brain. Opt Lett.

[55] Wolf M, Franceschini M, Paunescu L, Toronov V, Michalos A, Wolf U, Gratton E, Fantini Sergio (2003). Absolute frequency-domain pulse oximetry of the brain: methodology and measurements. Adv Exp Med Biol.

[56] Vetter R, Rossini L, Ridolfi A, Sola J, Chetelat O, Correvon M, Krauss J (2009). Frequency Domain SpO2 Estimation Based on Multichannel Photoplethysmographic Measurements at the Sternum.

[57] Zhao Y, Applegate MB, Istfan R, Pande A, Roblyer D (2018). Quantitative real-time pulse oximetry with ultrafast frequency-domain diffuse optics and deep neural network processing. Biomed Opt Express.

[58] Fantini S, Franceschini MA, Fishkin JB, Barbieri B, Gratton E (1994). Quantitative determination of the absorption spectra of chromophores in strongly scattering media: a light-emitting-diode based technique. Appl Opt.

[59] Fantini S, Hueber D, Franceschini MA, Gratton E, Rosenfeld W, Stubblefield PG, Maulik D, Stankovic MR (1999). Non-invasive optical monitoring of the newborn piglet brain using continuous-wave and frequency-domain spectroscopy. Phys Med Biol.

[60] Fantini S, Franceschini MA, Gratton E (1994). Semi-infinite-geometry boundary problem for light migration in highly scattering media: a frequency-domain study in the diffusion approximation. J Opt Soc Am B.

[61] Kleiser S, Nasseri N, Andresen B, Greisen G, Wolf M (2016). Comparison of tissue oximeters on a liquid phantom with adjustable optical properties. Biomed Opt Express.

[62] Stankovic M, Maulik D, Rosenfeld W, Stubblefield P, Kofinas A, Drexler S, Nair R, Franceschini M, Hueber D, Gratton E, Fantini S (1999). Real-time optical imaging of experimental brain ischemia and hemorrhage in neonatal piglets. J Perinat Med.

[63] Fantini S, Franceschini MA, Gratton E, Hueber D, Rosenfeld W, Maulik D, Stubblefield PG, Stankovic MR (1999). Non-invasive optical mapping of the piglet brain in real time. Opt Express.

[64] Hallacoglu B, Sassaroli A, Wysocki M, Guerrero-Berroa E, Schnaider Beeri M, Haroutunian V, Shaul M, Rosenberg I, Troen A, Fantini S (2012). Absolute measurement of cerebral optical coefficients, hemoglobin concentration and oxygen saturation in old and young adults with near-infrared spectroscopy. J Biomed Opt.

[65] Kleiser S, Ostojic D, Andresen B, Nasseri N, Isler H, Scholkmann F, Karen T, Greisen G, Wolf M (2018). Comparison of tissue oximeters on a liquid phantom with adjustable optical properties: an extension. Biomed Opt Express.

[66] SOMANETICS INVOS 3100A CEREBRAL OXIMETER (INVOS). U.S. Food and Drug Administration (FDA) 510(k) Database.

[67] FORE-SIGHT ABSOLUTE CEREBRAL AND SOMATIC OXIMETER, MC 2000 SERIES, MODELS MC2000, MC2010, MC2020, MC2030. U.S. Food and Drug Administration (FDA) 510(k) Database.

[68] Franceschini MA, Thaker S, Themelis G, Krishnamoorthy KK, Bortfeld H, Diamond SG, Boas DA, Arvin K, Grant PE (2007). Assessment of infant brain development with frequency-domain near-infrared spectroscopy. Pediatr Res.

[69] Zhang Z, Bolz N, Laures M, Oremek M, Schmidt C, Qi M, Khatami R (2017). Cerebral blood volume and oxygen supply uniformly increase following various intrathoracic pressure strains. Sci Rep.

[70] Zhang Z, Schneider M, Laures M, Qi M, Khatami R (2016). The comparisons of cerebral hemodynamics induced by obstructive sleep apnea with arousal and periodic limb movement with arousal: a pilot NIRS study. Front Neurosci.

[71] Zhang Z, Khatami R (2014). Predominant endothelial vasomotor activity during human sleep: a near-infrared spectroscopy study. Eur J Neurosci.

[72] Cleveland WS, Devlin SJ (1988). Locally weighted regression: an approach to regression analysis by local fitting. Journal of the American Statistical Association.

[73] Watzman H, Kurth C, Montenegro L, Rome J, Steven J, Nicolson S C (2000). Arterial and venous contributions to near-infrared cerebral oximetry. Anesthesiology.

[74] Abay TY, Kyriacou PA (2017). Photoplethysmography for blood volumes and oxygenation changes during intermittent vascular occlusions. J Clin Monit Comput.

[75] REGIONAL OXIMETER. U.S. Food and Drug Administration (FDA) 510(k) Database.

[76] INSPECTRA TISSUE SPECTROMETER SYSTEM (INSPECTRA), MODEL 325. U.S. Food and Drug Administration (FDA) 510(k) Database.

[77] VIOPTIX ODISSEY TISSUE OXIMETER TISSUE OXIMETER, MODEL OXY-2. U.S. Food and Drug Administration (FDA) 510(k) Database.

[78] Benni PB, MacLeod D, Ikeda K, Lin H (2018). A validation method for near-infrared spectroscopy based tissue oximeters for cerebral and somatic tissue oxygen saturation measurements. J Clin Monit Comput.

[79] Kreeger RN, Ramamoorthy C, Nicolson SC, Ames WA, Hirsch R, Peng LF, Glatz AC, Hill KD, Hoffman J, Tomasson J, Kurth CD (2012). Evaluation of pediatric near-infrared cerebral oximeter for cardiac disease. Ann Thorac Surg.

[80] Bickler PE, Feiner JR, Rollins MD (2013). Factors affecting the performance of 5 cerebral oximeters during hypoxia in healthy volunteers. Anesth Analg.

[81] Henson L, Calalang C, Temp J, Ward D S (1998). Accuracy of a cerebral oximeter in healthy volunteers under conditions of isocapnic hypoxia. Anesthesiology.

[82] Shah N, Trivedi NK, Clack SL, Shah M, Shah PP, Barker S (2000). Impact of hypoxemia on the performance of cerebral oximeter in volunteer subjects. J Neurosurg Anesthesiol.

[83] Benni P, Chen B, Dykes F, Wagoner S, Heard M, Tanner A, Young T, Rais-Bahrami K, Rivera O, Short L (2005). Validation of the CAS neonatal NIRS system by monitoring vv-ECMO patients: preliminary results. Adv Exp Med Biol.

[84] Rais-Bahrami K, Rivera O, Short BL (2006). Validation of a noninvasive neonatal optical cerebral oximeter in veno-venous ECMO patients with a cephalad catheter. J Perinatol.

[85] Ikeda K, MacLeod DB, Grocott HP, Moretti EW, Ames W, Vacchiano C (2014). The accuracy of a near-infrared spectroscopy cerebral oximetry device and its potential value for estimating jugular venous oxygen saturation. Anesth Analg.

[86] Redford D, Paidy S, Kashif F (2014). Absolute and trend accuracy of a new regional oximeter in healthy volunteers during controlled hypoxia. Anesthesia & Analgesia.

[87] Franceschini MA, Boas DA, Zourabian A, Diamond SG, Nadgir S, Lin DW, Moore JB, Fantini S (2002). Near-infrared spiroximetry: noninvasive measurements of venous saturation in piglets and human subjects. J Appl Physiol (1985).

[88] MacDonald MJ, Tarnopolsky MA, Green HJ, Hughson RL (1999). Comparison of femoral blood gases and muscle near-infrared spectroscopy at exercise onset in humans. J Appl Physiol (1985).

[89] Lee H, Ko H, Lee J (2016). Reflectance pulse oximetry: practical issues and limitations. ICT Express.

[90] Tamura T (2019). Current progress of photoplethysmography and SPO for health monitoring. Biomed Eng Lett.

[91] Mendelson Y, Ochs B (1988). Noninvasive pulse oximetry utilizing skin reflectance photoplethysmography. IEEE Trans Biomed Eng.

[92] Hickey M, Kyriacou PA (2007). Optimal spacing between transmitting and receiving optical fibres in reflectance pulse oximetry. J Phys: Conf Ser.

[93] Pittman S, Ayas N, MacDonald M, Malhotra A, Fogel R, White P (2004). Using a wrist-worn device based on peripheral arterial tonometry to diagnose obstructive sleep apnea: in-laboratory and ambulatory validation. Sleep.

